# Presynaptic maturation of inhibitory connections onto vasoactive intestinal polypeptide-expressing GABAergic interneurons in the mouse barrel field

**DOI:** 10.1007/s00424-025-03101-8

**Published:** 2025-06-25

**Authors:** Clara A. Simacek, Sergei Kirischuk, Thomas Mittmann

**Affiliations:** Institute for Physiology, University Medical Centre of the Johannes Gutenberg University Mainz, Mainz, Germany

**Keywords:** VIP, Interneuron, Development, Inhibition, Somatosensory, Quantal analysis

## Abstract

Vasoactive intestinal polypeptide-expressing inhibitory interneurons (VIP-INs) in the adult barrel cortex are crucial for mediating active whisking (AW) by disinhibiting pyramidal neurons. Past studies have investigated the development of VIP-IN network integration, focusing mainly on the excitatory network or the postsynaptic side of the inhibitory network. Hence, we aimed to explore the inhibitory network integration of VIP-INs, concentrating on the presynaptic side. We addressed this by investigating VIP-INs in three different age groups (postnatal day (P)8–P10, P14–P16, and P30–P36) in *Vip-IRES-cre x tdTomato* mice with whole-cell patch clamp recordings. By placing a stimulation electrode into L4 of the barrel field, we elicited electrically-evoked inhibitory postsynaptic currents (eIPSCs) in L2/3 VIP-INs following a high-frequency stimulation. We then analysed recorded eIPSCs by applying the binomial model of synaptic transmission. Our results show significant increases in both the number of readily-releasable vesicles and the presynaptic release probability between P9 and P15, suggesting that the inhibitory network integration is at least partially conducted via a presynaptic functional maturation. Despite an increase in the release probability, synaptic depression is decreased at P30–P36 due to an accelerated vesicle replenishment rate within the same time window. Lastly, asynchronous vesicle release decreases in favour of a stimulus-locked signal transmission by P30–P36. Our results suggest a maturation of the inhibitory projections towards a strong, precise, and stimulus-locked inhibition. This can be physiologically relevant to define the temporal precision of AW at the relevant frequencies.

**Supplementary Information:**

The online version contains supplementary material available at 10.1007/s00424-025-03101-8.

## Introduction

Inhibitory signal transmission is indispensable to dynamically guide and process excitatory information flow through an organism’s central nervous system [1–3]. In the cerebral neocortex, its main cellular substrates are local inhibitory interneurons (INs), which release the neurotransmitter γ-amino-butyric-acid (GABA) [4, 5]. Even though GABAergic INs comprise only about 15–20% of all cerebral neurons, they show a high heterogeneity in a variety of features (e.g., morphology, electrophysiological profile, molecular marker expression) and it is the subject of current research to categorise them into distinct subpopulations [6–9].

One of these subpopulations is classified based on the expression of the ionotropic serotonin receptor 5HT3aR and can be further subdivided into neurons coexpressing the vasoactive intestinal polypeptide (VIP) [10, 11]. Cerebral VIP-positive INs comprise about 15% of all GABAergic INs in the mouse brain and represent a heterogeneous subpopulation of neurons, as they can be further subclassified based on, e.g., their morphology, electrophysiological properties, the expression of markers such as calretinin or cholecystokinin, and their preferred downstream targets [12]. Interestingly, Acsády et al. [13] and Gulyás et al. [14] first described that a subpopulation of VIP-expressing INs is recruited in a circuit motif referred to as disinhibition. In this circuitry, VIP-positive INs preferentially inhibit somatostatin (SST)-expressing INs (approx. 60%), and by this, downstream pyramidal neurons are released from their previous inhibition mediated by the SST-INs [15–19]. In the adult murine somatosensory cortex, VIP-positive interneuron-mediated disinhibition is involved in the execution of active whisking (AW) [19, 20]. For this, VIP-expressing INs of the barrel field receive long-range excitatory projections from the primary motor cortex (M1) [19, 21, 22]. In addition, the main inhibitory inputs of supragranular VIP-positive INs are from local interneurons. Here, parvalbumin (PV)-expressing INs mainly target the perisomatic regions of VIP-positive INs, while SST-positive INs preferentially contact the distal-dendritic regions of VIP cells [23, 24]. Additionally, especially PV-INs, which are most abundant in L4 of the barrel field, are known to mediate feedforward inhibition to supragranular neurons by receiving excitation from the ventral posteriomedial nucleus (VPM) of the thalamus [20, 24–26].

Mice start to actively use their whiskers at around postnatal day (P)14 [27–29] and recent studies investigated the involvement of VIP-positive INs in the context of the developmental emergence of AW. So far, the main focus has been on the excitatory signal transmission. For instance, before P14, VIP-positive INs receive direct excitatory input from the sensory thalamus, which is strongly weakened around the onset of AW [30]. Moreover, the beforementioned excitatory projections from M1 were reported to exist already at P5 [31], which is in line with our data showing that these projections are functional from P8 on [32]. In contrast to the excitatory projections, the development of the inhibitory projections towards VIP-positive INs remains not fully investigated.

Our aforementioned study not only investigated the excitatory network integration of VIP-positive INs into the barrel field but also inspected the integration into the inhibitory network. Here, results mainly pointed to developmental changes on the postsynaptic side. For instance, the obtained findings showed a strengthening and acceleration of GABAergic currents, which may reflect an increase in the expression of GABA_A_-receptors and a putative change in their subunit composition around the onset of AW [32]. These developmental changes may be beneficial to provide a strong and timely precise inhibitory signal transmission to VIP-positive INs.

With the present study, we want to further elucidate the development of the inhibitory signal transmission provided to VIP-positive INs by focusing on putative maturational processes on the presynaptic side. For this, we used a *Vip-IRES-cre x tdTomato* reporter mouse line and performed immunohistological and electrophysiological investigations on VIP-positive interneurons in L2/3 of the barrel field (VIP-INs) at three different age groups: P8–P10, e.g. before the onset of AW; P14–P16, e.g. during the onset of AW; and P30–P36, e.g. in adult animals. For our electrophysiological experiments in particular, we performed whole-cell patch clamp recordings and analysed electrically evoked inhibitory postsynaptic currents in the frame of the binomial model of synaptic transmission [33].

Our results yield that the inhibitory network integration partially takes place via a functional maturation on the presynaptic side, as reflected by an increase in the number of readily-releasable vesicles. We show that the presynaptic release probability increases between the P8–10 and P14–16 age groups; however, a consequently resulting synaptic depression is avoided by an accelerated vesicle recycling rate when reaching adulthood. Additionally, the inhibitory projections undergo a maturation from an asynchronous mode of vesicle release towards a stimulus-locked signal transmission when reaching adulthood.

We further discuss how this overall increase in the precision of inhibition may convey a pivotal role in firstly defining temporal integration windows and secondly in performing AW in the appropriate frequencies.

## Materials and methods

### Animals and ethical statement

This study utilised homozygous *Vip-IRES-cre* mice (Vip^tm1(cre)Zjh^, JAX Stock #031628, Jackson Laboratory, Bar Harbor, USA) crossed with homozygous tdTomato reporter mice (*Ai14*,* B6.Cg-Gt(ROSA)26Sor tm14(CAG-tdTomato)Hze/J*, JAX Stock # 007914, The Jackson Laboratory, [34]) to generate *Vip-IRES-cre x tdTomato* offspring. Experiments were conducted on these mice at three postnatal time points: P8–P10 (labelled as P9, *N* = 11: 6 female, 2 male, 3 not indicated), P14–P16 (labelled as P15, *N* = 10: 1 female, 7 male, 2 not indicated), and P30–P36 (labelled as P30 +, *N* = 12: 3 female, 9 male). Animals were housed under standard laboratory conditions with a 12-h light/dark cycle, free access to food and water, and a constant temperature of 23 ± 2 °C. All experimental procedures were designed to minimise the number of animals used, and they were performed following German and European animal welfare regulations (2010/63/EU).

### Slice preparation

Animals were deeply anaesthetised by isoflurane (4%) inhalation, before they were decapitated, and their brains were immediately immersed in ice-cold, oxygenated (95% O_2_, 5% CO_2_) cutting artificial cerebrospinal fluid (cACSF; composition in mM: 87 NaCl, 37.5 choline chloride, 2.5 KCl, 7 MgSO4, 0.5 CaCl2, 1.25 NaH2PO4, 25 NaHCO3, 25 D-glucose; pH 7.4). 300µm thick coronal slices were obtained using a vibratome (VT1200 S, Leica, Wetzlar, Germany). Slices containing somatosensory areas were incubated in cACSF at 37 °C for 20 min and then transferred to regular ACSF (composition in mM: 125 NaCl, 2.5 KCl, 1 MgSO4, 2 CaCl2, 1.25 NaH2PO4, 25 NaHCO3, 25 D-glucose; pH 7.4) at room temperature for at least 45 min before use. If not otherwise indicated, all reagents were obtained from Carl Roth, Karlsruhe, Germany.

### Whole-cell patch clamp recordings

Whole-cell patch clamp recordings were obtained from visually identified VIP-positive interneurons in layers 2/3 (L2/3) of the primary somatosensory cortex (VIP-INs). Brain slices were placed in a submerged recording chamber on an Olympus BX51WI upright microscope (Olympus, Tokyo, Japan) and continuously perfused with oxygenated ACSF maintained at 37 °C. The barrel field of the somatosensory cortex was optically identified using a 5 × objective (Olympus). VIP-INs, expressing tdTomato fluorescence, were then selected for electrophysiological recordings using a 40 × objective (Olympus) and a fluorescent light source (U-M49010, Olympus) equipped with a tdTomato filter cube (U-M49010, Olympus). In this study, recordings were mainly performed on VIP-expressing neurons located in L2/3 of the barrel field, specifically near L1.

Recordings were performed using an Axopatch-200B amplifier and Clampex 11.2 software (Molecular Devices, San José, CA, USA). Recording pipettes were fabricated from borosilicate glass capillaries (GB 150F-8P; Science Products, Frankfurt, Germany) using a DMZ Zeitz-Puller (Planegg, Germany). Pipettes were filled with a high Cl^−^ intracellular solution containing (in mM): 130 KCl, 5 NaCl, 5 EGTA, 20 HEPES, 0.5 CaCl2, 2 Mg-ATP, 0.3 Na-GTP; pH 7.3 adjusted with KOH. For measuring electrically-evoked inhibitory postsynaptic currents (eIPSCs), N-ethyl lidocaine (QX-314, 2.5 mM) was added to block voltage-gated Na^+^ channels of the patched cell. All pipettes had resistances ranging from 3 to 10 MΩ and lower resistances were preferred for older animals. Serial resistance (R_s_) and membrane resistance (R_m_) were continuously monitored, and cells with R_s_ exceeding 35 MΩ were discarded. Data were acquired using a Digidata-1400 digitizer and Clampex 11.1 software (Molecular Devices), filtered at 2 kHz, and sampled at 50 kHz.

#### Miniature inhibitory postsynaptic currents

Miniature inhibitory postsynaptic currents (mIPSCs) were measured by using a high Cl^−^ intracellular solution (see above). To inhibit excitatory signal transmission, 6,7-dinitroquinoxaline-2,3-dione (DNQX, 20 µM) and 2-amino-5-phosphonovalerinans acid (DAP-5, 25 µM) were added to the ACSF, as well as tetrodotoxin (TTX, 1 µM) to block voltage-gated Na^+^-channels. R_s_ compensation was not applied, and after achieving the whole-cell configuration, mIPSCs were recorded at a holding potential of − 70 mV for 2 min. mIPSCs were analysed using MiniAnalysis software (Synaptosoft, Fort Lee, NJ, USA).

#### Electrical stimulation

eIPSCs were recorded from L2/3 VIP-INs using a high chloride intracellular solution (see above) and adding QX-314 (2.5 mM), while holding the cells at − 70 mV. A bipolar tungsten stimulating electrode was positioned in L4 of the barrel field. Excitatory synaptic transmission was blocked by bath application of DNQX (20 µM) and DAP-5 (25 µM) to the ACSF. Maximum stimulation intensity was determined by applying 20 ns electrical pulses (A360 stimulus isolator; World Precision Instruments, USA) ranging from 25 to 500 µA until the eIPSC amplitude saturated (typical intensity: 300–400 µA) [32]. A high-frequency stimulation protocol, consisting of 20 pulses at 20 Hz, was applied, with at least three repetitions. To avoid the induction of synaptic plasticity, the stimulation protocol was applied only once per minute. eIPSC data were analysed offline using PeakCount software v3.2, which employs a derivative-crossing algorithm for detection and visual inspection of postsynaptic currents.

### Analysis of electrically evoked postsynaptic responses

Analysis of eIPSCs following a high-frequency stimulation was conducted in the frame of the binomial model of synaptic transmission [33]. This model relies on the following four assumptions: (i) there is a constant number of release sites (*N*_*syn*_) at which vesicles are released with an average release probability *p*_*r*_; (ii) in response to an action potential, each synaptic release site releases either none or at least one vesicle; (iii) a single vesicle evokes an invariant, quantal IPSC *q*; and (iv) the synaptic release sites are independent from each other. Under these presumptions, mean eIPSC amplitudes are given by:

1 $$mean\; eIPSC\; amplitude = {N}_{syn}{p}_{r}q$$

The three quantal parameters *q*, *N*_*syn*_, and* p*_*r*_ were determined in the following way:

Firstly, IPSCs that appeared after the termination of the high-frequency stimulation, namely delayed IPSCs (dIPSCs), were used to obtain the quantal size *q* [35]. dIPSCs represent spontaneous IPSCs (sIPSCs) since TTX was absent in the ACSF, and they were recorded within the first 4 s after the termination of the high-frequency stimulation. As shown by Simacek et al. [32], the sIPSC rate does not exceed 3 Hz in all age groups, so a putative contribution of sIPSCs originating from non-stimulated synaptic contacts was neglected. For each cell, the mean dIPSC amplitude was taken as an estimation of *q* (Fig. 2C). Additionally, the mean dIPSC decay time was determined by applying a mono-exponential fit. The product of the mean dIPSC amplitude and its decay time was taken to model the quantal charge transfer *q*_*c*_ [35].

Secondly, the calculation of the number of synaptic release sites *N*_*syn*_ required to first estimate the readily-releasable pool (RRP) size. For this, the amplitudes of stimulus-locked eIPSCs during the high-frequency stimulation protocol were measured. eIPSCs were considered stimulus-locked when they were elicited within a time interval of less than 3.5 ms following a stimulus. Estimation of the RRP follows the assumption that upon a high-frequency stimulation, (1) the RRP becomes depleted, (2) stimulus-locked eIPSCs after RRP depletion are supported only by vesicle recycling, meaning the replenishment of the RRP, and (3) the replenishment rate is constant [35, 36]. We have plotted the cumulative eIPSC amplitude as a function of the pulse number, i.e., time (Fig. 2A). After RRP depletion, the cumulative eIPSC amplitude rises linearly and its slope is dependent on the replenishment rate. Under this assumption, a linear fit to the cumulative eIPSC amplitude plot was applied over the last 10 pulses. By back-extrapolating the linear fit to the onset of stimulation, the resting RRP [nA] can be estimated. Following a normalisation of the RRP [nA] to *q* gives an estimation of the RRP [n vesicles], which is equal to *N*_*syn*_ in the frame of the binomial model of synaptic transmission.

Thirdly, Eq. (1) was converted to calculate the release probability *p*_*r*_:

2 $${p}_{r } = \frac{eIPSC}{{N}_{syn} q} = \frac{eIPSC}{RRP}$$

The slope of the cumulative eIPSC histogram used for RRP estimation represents the presynaptic vesicle recycling rate. This parameter measured in nAs depends on the RRP size (*N*_*syn*_) and quantal size (*q*). To obtain the mean replenishment rate at a single release site, the replenishment rate measured in nAs was normalised to *N*_*syn*_ and *q* (Fig. 3A).

Calculation of synchronous and asynchronous vesicle release was conducted in the following way: First, all traces of evoked responses following the high-frequency stimulation were averaged per cell. Next, the baseline was set at pre-stimulation levels and the area of the last response was calculated. More specifically, this means that the integral between the peak of the last response and when the trace reached the baseline again was calculated. This was referred to as area_total_ and was expressed in pA*ms (Fig. 4C). Area_total_ consists of both synchronous and asynchronous vesicle release (SR and ASR, respectively). To calculate the ASR, SR was subtracted from area_total_. We modelled the SR by multiplying the amplitude of the last evoked event by the decay time constant of dIPSCs, the latter therefore serving as the quantal decay time constant. After subtracting the SR from area_total_, the result represented the ASR in pA*ms. To express it in n vesicles, we normalise it to *q*_c_. For calculation the contribution of the ASR to area_total_, the latter was also normalised to *q*_*c*_, so that the ASR/area_total_ ratio could be calculated.

### Immunohistochemistry

Following deep anaesthesia with isoflurane (4%; AbbVie, Wiesbaden, Germany), mice (P9, P15, and P30 +) were decapitated, and their brains were fixed overnight in 4% PFA at 4 °C. After rinsing in phosphate buffer (PB), brains were placed in 30% sucrose in 0.1 PB for 24 h and sectioned into 50 µm on a freezing microtome (CM 1325; Leica Microsystems, Wetzlar, Germany). For free-floating staining, coronal trimmed slices of the somatosensory cortex were washed with phosphate-buffered saline (PBS) 0.01 M and then blocked and permeabilised for 2 h at room temperature (RT) with a solution containing 7% normal donkey serum (017–000–121, Dianova, Hamburg, Germany) and 0.8% Triton X-100 in 0.01 M PBS. The sections were incubated for 2 days at 4 °C in the primary antibodies (ABs) (guinea pig) anti-VGAT 1:500 (131004, Synaptic Systems, Göttingen, Germany) and (goat) anti-mCherry 1:100 (AB0040-200, Sicgen, OriGene Technology GmbH, Herford, Germany) in a staining buffer containing 2% bovine serum albumin (001–000–161, Jackson ImmunoResearch, Dianova, Germany) with 0.05% azide and 0.3% Triton X-100 in 0.01 M PBS. Slices were washed extensively with 0.01 M PBS and then incubated with the appropriate secondary antibodies and 0.5 μg/mL DAPI (A4099.0005, AppliChem, Darmstadt, Germany) for 2 h at RT in a staining solution containing 2% bovine serum albumin (001–000–161, Dianova) with 0.05% azide. The secondary antibodies were Alexa Fluor® 647 AffiniPure™ F(ab')₂ Fragment Donkey Anti-Guinea Pig IgG (H + L) 1:200 (706–606-148, Jackson ImmunoResearch, Dianova, Germany) and Cy™3 AffiniPure™ Donkey Anti-Goat IgG (H + L) 1:400 (705–, Jackson ImmunoResearch, Dianova, Germany). After washing in 0.01 M PBS, slices were mounted onto slides and coverslipped with Fluoromount-G (SouthernBiotech, Birmingham, USA) mounting medium.

### Confocal imaging and image processing

Using a confocal microscope (Zeiss LSM 710, Carl Zeiss AG, Oberkochen, Germany), L2/3 of the barrel field was visually identified based on neuroanatomical landmarks in the DAPI signal at a × 25 magnification. Since the border between L1 and L2/3 was used as a neuroanatomical hallmark, the majority of imaged cells were located in the upper layers of L2/3. In a 63 x or 100 x magnification, VIP-INs were first identified based on their tdTomato expression. Then, a z-stack of three images was recorded at the maximum circumference of the soma of the identified VIP-IN. Z-stacks had a total optical thickness of 0.84 µm (0.42 µm image interval within every z-stack) for all three channels (DAPI, tdTomato, VGAT). 80 - 99 cells were imaged per age group. From the z-stacks, maximum intensity projections were generated and further analysed in Fiji ImageJ software. A custom-written macro generated a region of interest (ROI) of the cell somas in the tdTomato channel. For this, a Gaussian blur with a radius of 8 µm was applied, followed by the Threshold function to generate binary masks. These were subsequently further adapted by the “Watershed” and “Fill Holes” functions to generate ROIs with smooth borders. To only keep the ROIs reflecting the shapes of cell somas, the function “Analyze Particles” with a minimum particle size of 50–75 µm^2^ was applied to filter out all smaller particles. The ROIs of VIP-IN somas were then plotted in the same position in the VGAT signal. Applying the “Multiplot” function returned a grey value profile along the line of the given ROI. The line width of the ROI was set to 5 pixels. Hence, VGAT puncta were detected when they fell in the line of the given ROI, which corresponds to 0.66 µm in the 63 × objective and 0.41 µm in the 100 × objective. Since the centre of the ROI line was aligned to the soma border, VGAT puncta were counted within a distance of 0.33 µm or 0.2 µm outside and inside of the soma border, depending on the respective objective. For each age group, one animal was imaged with the 100 × objective and 2 animals were imaged with the 63 × objective.

A custom-written MATLAB (version R 2023a, MathWorks, Natick, MA, USA) code was used to further analyse the grey value profiles. The code first calculated the standard deviation (SD) of the given grey value profile and set a threshold at the 2.5-fold SD for each cell and age group. All areas between the grey value trace and the threshold were calculated and filtered out if they were smaller than a defined area. The remaining areas were counted and set into relation to the length of the circumference of the corresponding ROI so that they were given as the number of VGAT puncta per 10 µm circumference.

### Data evaluation and statistics

Data were analysed in Microsoft Excel 2021 and GraphPad Prism (version 9.2.0, San Diego, CA, USA). Results are reported as mean ± SEM. Normality and homoscedasticity were assessed using Shapiro–Wilk and Bartlett’s tests, respectively. One-way ANOVA was used when data met these assumptions and a post hoc Tukey’s test was applied for multiple comparisons. If one or none of the criteria was met, the non-parametric Kruskal–Wallis test with Dunn’s post hoc test for multiple comparisons was applied. Statistical significance is indicated as *p < 0.05, **p < 0.01, ***p < 0.001, and ****p < 0.0001.

## Results

### Inhibitory network integration of VIP-INs in the developing barrel cortex

**Fig. 1:**
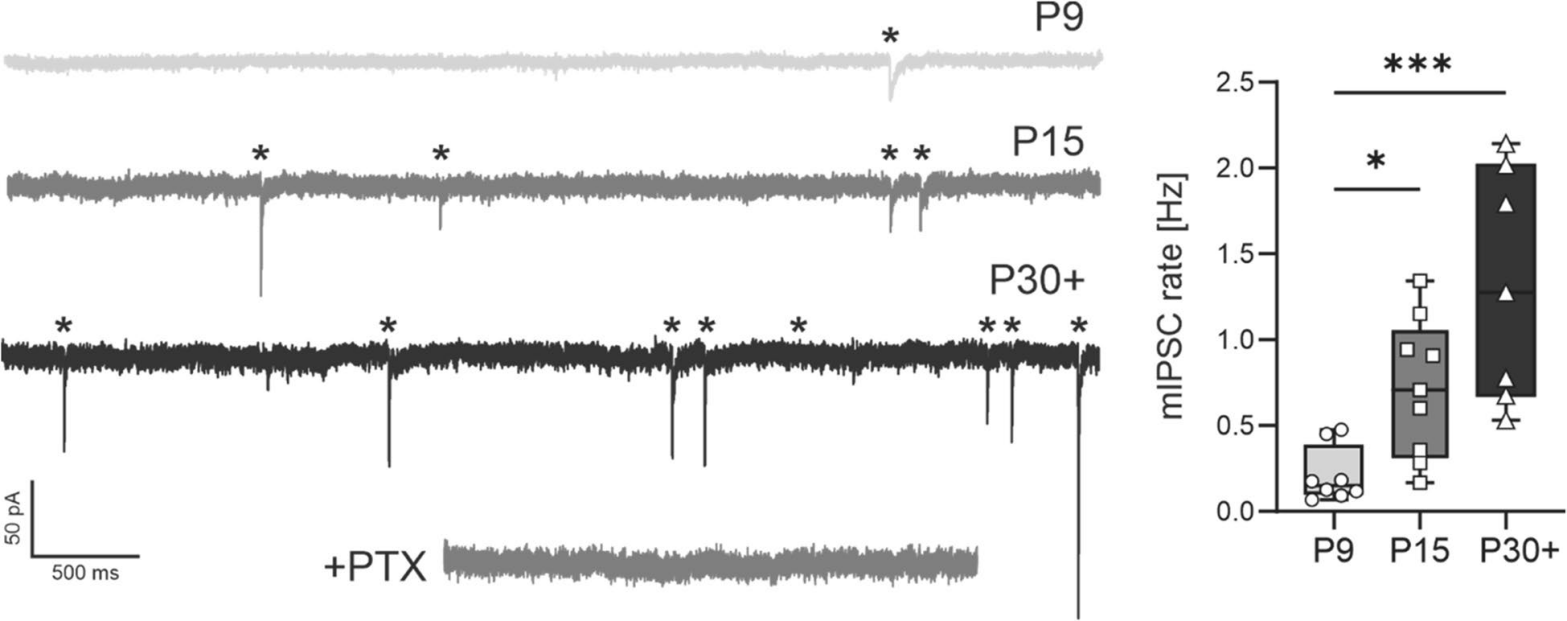
Increase in mIPSC rate indeveloping VIP-INs. Left: representative traces of mIPSCs recorded at P9, P15, and P30 +. Bath application of 50 µM PTX completely abolished mIPSCs. Right: box plots indicating a significant increase in mIPSC rate between P9 and P15. Corresponding *p*-values: *p* = 0.0439 (P9 and P15) and *p* = 0.0009 (P9 and P30 +), Kruskal–Wallis. Number of cells: 10–12. *p < 0.05, ***p < 0.001. Box plots indicating the minimum and maximum value, median, and 25% and 75% percentiles

In our recent study, we demonstrated that VIP-INs become integrated into the inhibitory network of the barrel field already between P9 and P15, which was indicated by a significant increase in the frequency or rate of recorded miniature inhibitory postsynaptic currents (mIPSCs) [32]. The traces in Fig. 1 illustrate reproduced mIPSC recordings at P9, P15, and P30 +. In general, an increase in the mPSC rate is suggested to reflect an increase in the number of synaptic contacts and/or an increase in the presynaptic release probability [37, 38].

To explore these two possibilities, we first assessed the number of putative inhibitory presynaptic structures as an estimate for the number of inhibitory synaptic contacts. For this, we performed immunostainings with an antibody against the vesicular GABA transporter (VGAT), a presynaptic marker for inhibitory synapses (Suppl. Figure 1,left). VGAT-positive puncta were counted along the somas of identified VIP-INs within a maximum distance of 0.66 µm as a quantification of putative presynaptic inhibitory structures. The number of VGAT puncta/10 µm significantly increased between P9 and P15 (Suppl. Figure 1, right). This indicates that the observed increase of the mIPSC rate between P9 and P15 might be partially due to an increase in putative presynaptic inhibitory structures, e.g. due to inhibitory synaptogenesis.

At GABAergic synapses, a single presynaptic action potential can induce the release of more than one vesicle, which is referred to as multiquantal release [38, 39]. Thus, the number of synaptic contacts may only give limited information about the functionality of the presynapses. Therefore, the terms release sites or number of vesicles will be used instead of synaptic contacts throughout the manuscript.

### Inhibitory release sites mature functionally on the presynaptic side

In addition to the developmental increase in the mIPSC rate, both the mean mIPSC amplitude and short-term plasticity of electrically-evoked IPSCs (eIPSCs) have been reported to demonstrate changes during the first 6 postnatal weeks [32]. Thus, we hypothesised that the inhibitory release sites undergo a presynaptic functional maturation, likely including an increase in the presynaptic release probability and in the number of readily-releasable vesicles.

**Fig. 2:**
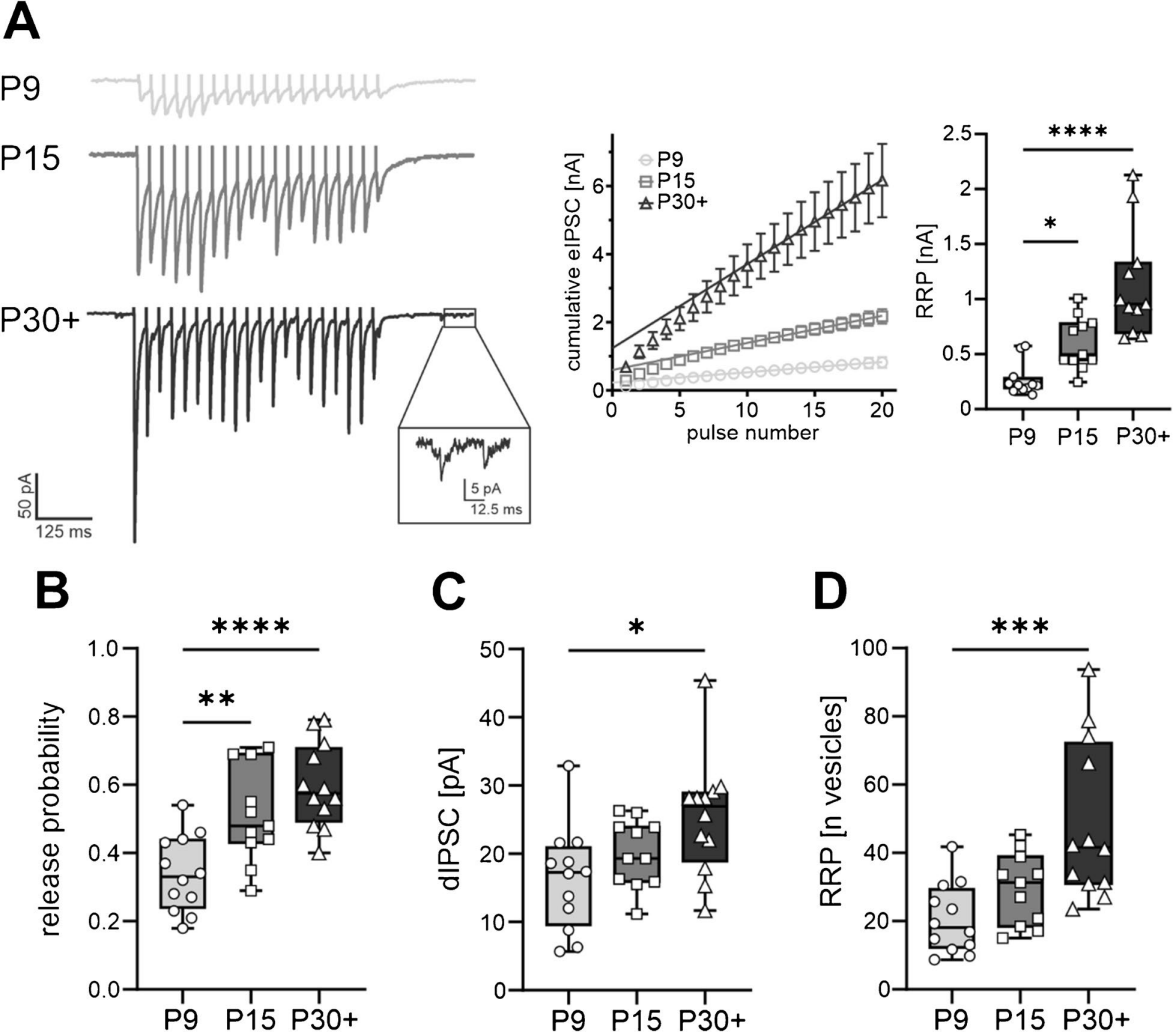
Functional presynaptic maturation of inhibitory connections onto VIP-INs. **A** Left: representative eIPSC traces elicited by a high-frequency electrical stimulation (20 pulses applied at 20 Hz) at P9, P15 and P30 +. Incision at the P30 + trace shows delayed IPSCs (dIPSCs) selected for the calculation of the quantal size for each cell. Middle: cumulative eIPSC amplitudes during a 20-Hz train. Lines indicate a linear fit of the last 10 events back-extrapolated to the onset of stimulation. The y-intercept serves as an estimation for the RRP. Right: box plots indicating that the RRP [nA] significantly increases between P9 and P15. Corresponding *p*-values: *p* = 0.0359 (P9 and P15) and *p* < 0.0001 (P9 and P30 +), Kruskal–Wallis. **B** Box plots indicating developmental changes in the presynaptic release probability. Corresponding *p*-values: *p* = 0.0051 (P9 and P15) and *p* < 0.0001 (P9 and P30 +), one-way ANOVA F(2,32) = 13.14. **C** Box plots indicating developmental changes in the quantal size (mean dIPSC amplitude) between P9 and P30 +; one-way ANOVA, F(2,32) = 4.727, *p* = 0.0132. **D** Box plots indicating developmental changes in the RRP (vesicles) between P9 and P30 +; Kruskal–Wallis, *p* = 0.0007. Number of cells: 11–12. *p < 0.05, **p < 0.01, ***p < 0.001, ****p < 0.0001. Box plots indicating the minimum and maximum value, median, and 25% and 75% percentile

To investigate this, we performed whole-cell patch clamp recordings on L2/3 VIP-INs of the barrel field at three different postnatal stages (P9, P15, and P30 +) and positioned a stimulation electrode in L4 of the barrel field. A high-frequency stimulation protocol (20 pulses at 20 Hz) was applied and synaptic responses were explored in the frame of the binomial model of synaptic transmission [33, 40] (see methods for details) (Fig. 2A left).

Firstly, we estimated the size of the readily-releasable pool (RRP) by calculating a cumulative eIPSC histogram out of the mean eIPSC amplitudes following the high-frequency stimulation. Next, we applied a linear fit to the last 10 values of the cumulative eIPSC histogram and back-extrapolated this to the stimulation onset. The RRP size represented in nA was obtained from the y-intercept of the linear curve.

The RRP size significantly increased between P9 and P15 from 0.3 ± 0.05 to 0.6 ± 0.07 nA (Kruskal–Wallis, *p* = 0.0359, Fig. 2A, right). With this, we could calculate the presynaptic release probability as a ratio of the amplitude of the first eIPSC to RRP size (see methods, Eq. 2). Interestingly, the significant increase in the mIPSC rate and VGAT puncta between P9 and P15 was accompanied by a significant increase in the presynaptic release probability within the same time window (Fig. 2B). Corresponding values are 0.3 ± 0.03 at P9 and 0.5 ± 0.04 at P15 (one-way ANOVA, F (2, 32) = 13.14, *p* = 0.0051).

The obtained RRP estimate represents a product of the number of release sites and the quantal amplitude. High-frequency stimulation depletes the RRP and leads to an asynchronous release of vesicles (delayed (d)IPSCs; see methods), presumably due to elevated presynaptic calcium concentrations ([Ca^2+^]) (Fig. 2A incision) [35]. The mean amplitude of dIPSCs was comparable to the mean mIPSC amplitude [32]. Similar to the mean mIPSC amplitudes, the mean dIPSC amplitude significantly increased by P30 + (Fig. 2 C, 16.2 ± 2.2 pA at P9 and 25.4 ± 2.5 pA at P30 +, respectively, one-way ANOVA, F (2,32) = 4.727, *p* = 0.0132), thus favouring the assumption that dIPSCs represent quantal events and can be taken as an estimate for *q* (see methods)*.* This indicates that the RRP, measured in nA, at least partially increases due to an increase in *q.* To estimate the number of vesicles in the RRP, e.g., the number of presynaptic release sites (see methods), we normalised the RRP [nA] to the quantal size *q*. This yielded a significant increase in the RRP [n vesicles] between P9 and P30 + from 20.1 ± 2.9 to 48.9 ± 6.7 vesicles (Kruskal–Wallis, *p* = 0.0007, Fig. 2D), showing that the RRP increases both due to an increase in the number of vesicles and an increase in the quantal size.

Taken together, the inhibitory network integration of VIP-INs in the barrel field takes place via an elevation of the presynaptic release probability and is presumably accompanied by an increase in the number of release sites. These developmental presynaptic changes can be beneficial to provide a strong and precise inhibitory signal transmission to VIP-INs.

### Faster vesicle replenishment rate compensates for presynaptic depression

**Fig. 3:**
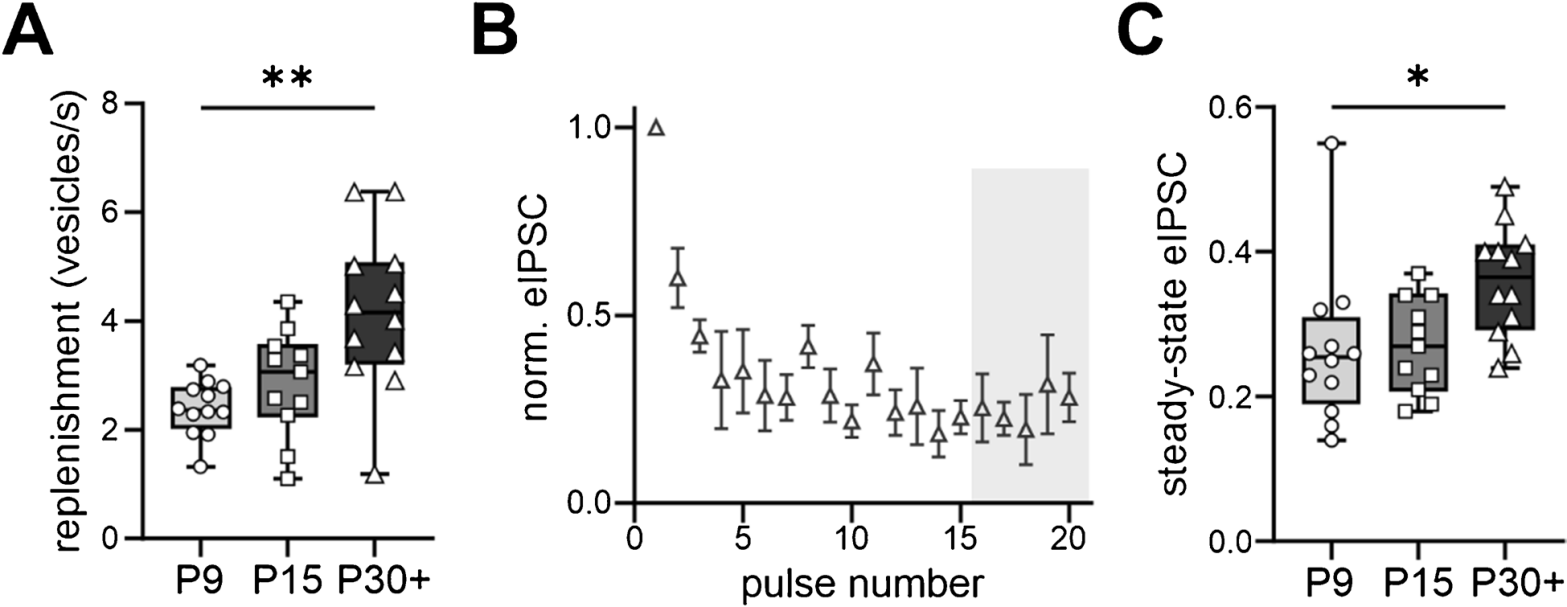
Decrease in presynaptic depression is mediated by an accelerated vesicle replenishment. **A** Vesicle replenishment rate becomes significantly accelerated between P9 and P30 +, *p* = 0.0013, Kruskal–Wallis. Vesicle replenishment rate was normalised to the RRP [nA] per cell. **B** Dependence of stimulus-locked eIPSC amplitude on the pulse number. eIPSCs were normalised to the first eIPSC of each cell. For determining the steady state, the average of the last 5 normalised eIPSCs was calculated (grey box). **C** Box plots showing a significant increase of the steady state between P9 and P30 +, *p* = 0.0115, Kruskal–Wallis. Number of cells: 11–12 cells. *p < 0.05, **p < 0.01. Box plots indicating the minimum and maximum value, median, and 25% and 75% percentiles

Although an increase in the RRP size is beneficial for a more precise inhibitory signal transmission at high activity levels, a higher release probability at P30 + could lead to a faster RRP depletion, thus eliminating the beforementioned increased precision. Therefore, we explored if the recycling rate of presynaptic vesicles (e.g. RRP replenishment) is developmentally regulated. For this, we used the slope of the cumulative eIPSC histogram to estimate the RRP replenishment rate. Since the slope depends on both the number of presynaptic release sites and the recycling rate, we normalised the slope to the RRP [nA] of each cell. As demonstrated in Fig. 3A, the replenishment rate became significantly faster between P9 and P30 + from 2.4 ± 0.1 to 4.2 ± 0.4 vesicles/s (Kruskal–Wallis, *p* = 0.0013).

As the replenishment rate of vesicles accelerates, a decreased synaptic depression during high-frequency stimulation should be expected. Hence, the synaptic depression was explored by normalising each eIPSC amplitude to the first eIPSC amplitude for every cell (Fig. 3B). The mean of the last 5 normalised eIPSC amplitudes was taken as a measure for the synaptic depression since they represent the replenishment at a fully emptied RRP (steady state). Figure 3C demonstrates that the mean steady-state eIPSC significantly increased between P9 and P30 + from 0.3 ± 0.03 to 0.4 ± 0.02 (Kruskal–Wallis, *p* = 0.0115).

These results indicate that the presynaptic inhibitory contacts on developing VIP-INs mature towards the ability to support a stimulus-locked vesicle release even at high activity levels.

### Maturation from a tonic towards a stimulus-locked inhibition provided to developing VIP-INs

In addition to stimulus-locked release, high-frequency stimulation may result in a desynchronised vesicle release, for instance, an asynchronous release (ASR) [40–42].

**Fig. 4:**
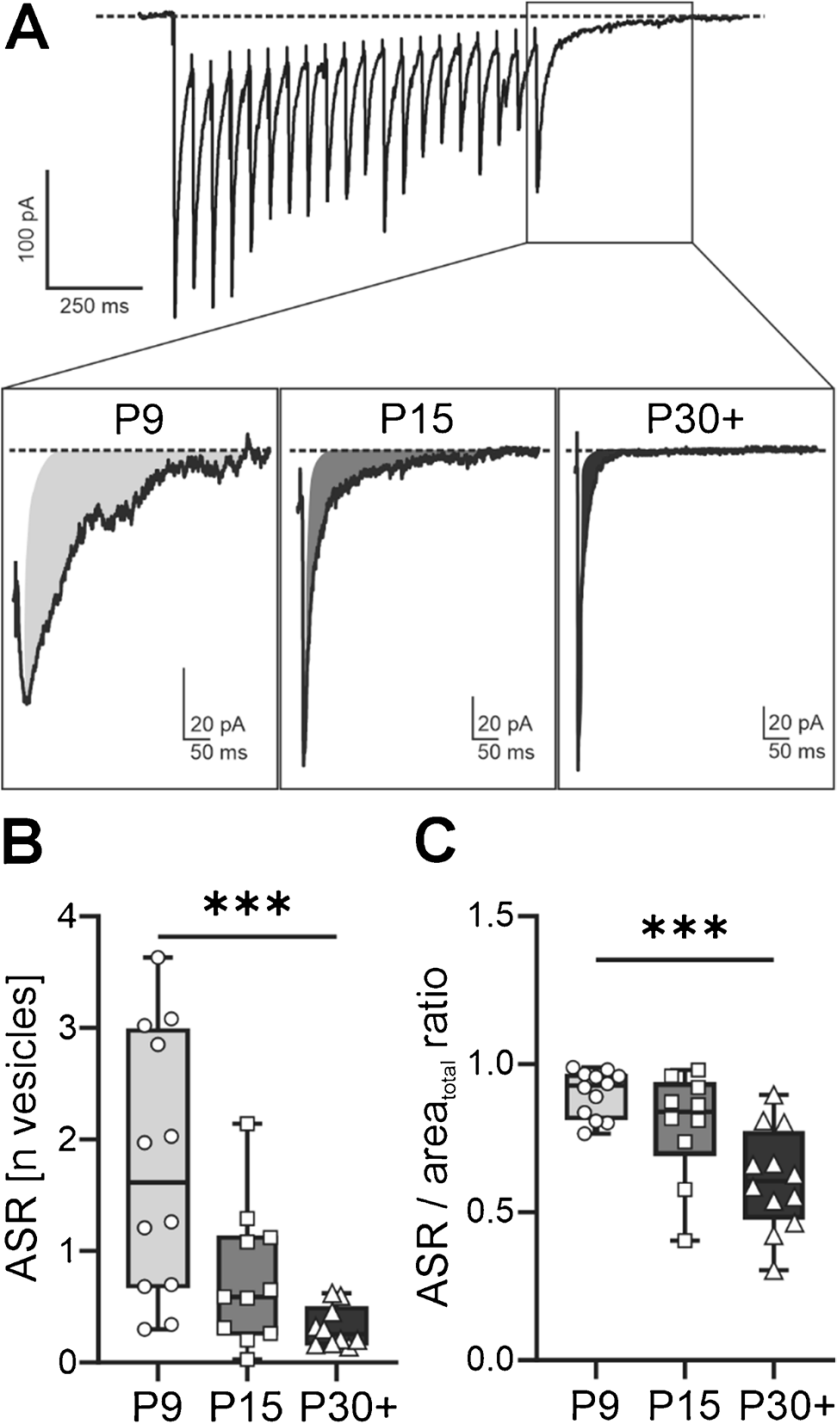
Inhibitory connections mature from an asynchronous towards a stimulus-locked release.** A** Visual representation of eIPSCs in response to a 20-Hz stimulation. Insets show the asynchronous release as grey highlighted areas at P9, P15, and P30 +. The areas for asynchronous release were normalised to the quantal size and RRP. **B** Box plots showing a significant reduction in the normalised asynchronous release between P9 and P30 +, *p* = 0.0007, Kruskal–Wallis. **C** Ratio of asynchronous release to the total area of the last evoked response gradually shifts towards synchronous release by P30 +, *p* = 0.0006, Kruskal–Wallis. Number of cells: 10–12. ***p < 0.001. Box plots indicating the minimum and maximum value, median, and 25% and 75% percentiles

To quantify this form of release, we calculated the area between the peak of the last evoked response (after termination of the electrical stimulations) and the point where it fully returned back to baseline (Fig. 4A). For the next step, the last evoked response was calculated in the absence of ASR, reflecting the synchronous release (SR). To model this, we calculated the area of the last response by multiplying its amplitude with the dIPSC decay time constant. This was done under the assumption that the last response in the absence of ASR follows a mono-exponential decay. To finally calculate the ASR, the area of the SR was subtracted from the overall area of the last response (Fig. 4A, incisions). For representing the ASR in numbers of vesicles at single release sites, the ASR was normalised to the product of the RRP and the quantal charge transfer *q*_*c*_. Statistical analysis revealed that the ASR significantly decreased between P9 and P30 + from 1.8 ± 0.3 to 0.3 ± 0.2 vesicles at single release sites (Kruskal–Wallis, *p* = 0.0007, Fig. 4B).

To explore the contribution of the ASR to the total area of the last evoked response, we first normalised the total area to the RRP and *q*_*c*_. Then, we divided the normalised ASR by the normalised total area and obtained the ASR/area_total_ ratio. The contribution of the ASR to the total area gradually decreased over development, which was statistically significant between P9 and P30 +. Corresponding values are 0.9 ± 0.02 for P9 and 0.6 ± 0.05 for P30 + (Kruskal–Wallis, *p* = 0.0006, Fig. 4C). This indicates that the inhibitory connections towards immature VIP-INs strongly support asynchronous vesicle release, presumably leading to rather tonic inhibition. As these connections mature, vesicle release becomes stimulus-locked and can mediate a temporally precise inhibitory signal transmission.

## Discussion

**Fig. 5:**
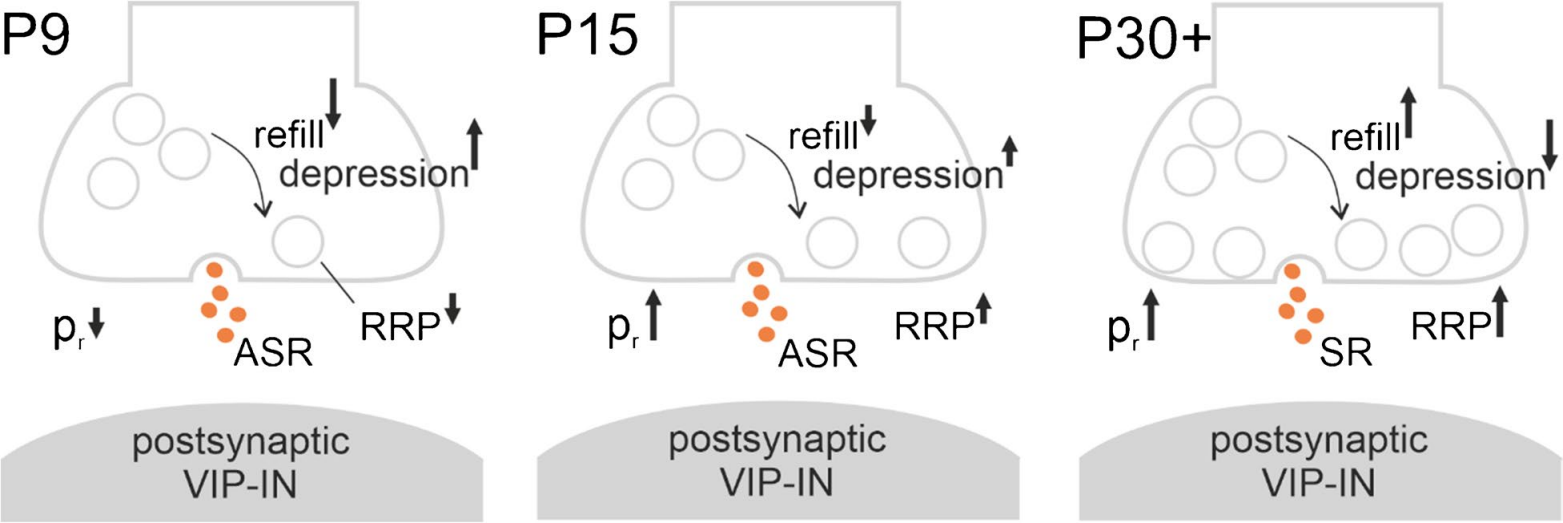
Summarising cartoon of maturing presynaptic inhibitory connections onto VIP-INs. The direction and length of the arrows indicate the direction and strength of parameter change at the respective time points. Between P9 and P15, the presynaptic release probability (p_r_) increases. The size of the readily releasable pool (RRP) increases gradually between P9 and P30 +. The vesicle refilling rate accelerates in parallel to a decrease in the synaptic depression when reaching P30 +. The mode of vesicle release is dominated by an asynchronous vesicle release (ASR) at P9 and P15, while it becomes more synchronous (SR) in the P30 + age group. These developmental changes are beneficial for mediating a strong and temporally precise inhibitory signal transmission towards VIP-INs

In this study, we investigated the presynaptic, developmental changes of inhibitory projections towards VIP-INs within the first 6 postnatal weeks. We report that (1) development of inhibitory inputs to VIP-INs inter alia takes place via changes of presynaptic functional properties, (2) both the number of releasable presynaptic vesicles and the presynaptic release probability are increased, (3) synaptic depression is compensated by an accelerated vesicle replenishment, and (4) the inhibitory connections mature from an asynchronous vesicle release towards a stimulus-locked signal transmission (see also Fig. 5).

### Inhibitory network integration depends on multiple processes, following different developmental trajectories

In our previous paper, we demonstrated a developmental increase in the mIPSC rate by P15 (Fig. 1, [32]). Now we provide experimental evidence that on the one hand this increase in mIPSC rate reflects a functional, presynaptic maturation. This was indicated by an increase in the presynaptic release probability by P15 and the number of functional release sites by P30 + (Fig. 2). On the other hand, our present data suggests that the mIPSC rate may additionally increase due to an increase in the number putative inhibitory presynaptic structures. This was demonstrated by an increase in VGAT-positive puncta in the same time window by P15 (Suppl. Figure 1).

Firstly, this increase in the presynaptic release probability is in agreement with a paired-pulse depression observed by P30 + [32]. Presynaptically, paired-pulse ratios (PPRs) are suggested to be inversely correlated to the release probability [43]. Interestingly, in contrast to the previous study [32], the release probability already increases by P15 in our present study. However, the PPR is modulated not only by the presynaptic site but might also depend on postsynaptic receptors [44]. As IPSCs in VIP-INs become faster during development [32], postsynaptic properties may additionally affect the PPR. Nevertheless, both of our measurements indicate an overall similar developmental trajectory of the release probability, namely that it increases when reaching adulthood. This suggests an increase in the synaptic strength of the inhibitory signal transmission towards VIP-INs. Interestingly, the quantal analysis revealed that distinct functional parameters undergo different developmental trajectories. For instance, the increase in the presynaptic release probability occurs by P15, while the RRP size/number of release sites gradually increases by reaching adulthood. This is in line with a study investigating the maturation of inhibitory projections from local inhibitory interneurons to L2/3 pyramidal neurons of the barrel field [45]. In addition, the same study demonstrates an increase in the mIPSC rate for the L2/3 pyramidal neurons in a similar time window to our results. This indicates that different cellular subtypes of the same location in the developing barrel field may undergo similar developmental trajectories in terms of the inhibitory network integration and presynaptic functional maturation. In addition, to determine the RRP expressed in number of vesicles, we estimated the quantal size *q* based on the mean dIPSC amplitude. In agreement with our previous paper, the mean dIPSC amplitudes were comparable to mIPSC amplitudes and both showed a developmental increase by reaching adulthood (Fig. 2, [32]). Therefore, using dIPSC amplitudes serves as a sufficient estimate for *q*, as also shown by different studies [40, 46, 47]. The developmental increase in *q* is also in line with other studies in the superior colliculus and in Cajal-Retzius cells [40, 48]. An increase in *q* can be due to different developmental changes in the pre- and postsynaptic side, such as the amount of neurotransmitters in the vesicle, number and kinetics of postsynaptic receptors, and—in case of GABA_A_-receptors—the electrochemical driving force of Cl^−^ and HCO_3_^−^ [37]. Since the RRP [nA] and *q* depend partially on postsynaptic parameters, normalisation of the RRP by *q* to the number of vesicles eliminates these falsifying factors and returns the RRP purely dependent on presynaptic parameters. Moreover, when applying the high-frequency stimulation, the [Ca^2+^] in the presynapse increases, leading to a desynchronised vesicle release after stimulus termination, which was chosen for estimating *q*. Since only the stimulated connections show an elevated [Ca^2+^], the majority of dIPSCs probably originate from the stimulated connection [35]. However, it cannot be excluded that some of the measured IPSCs are spontaneously released from other unstimulated connections (sIPSCs). Our previous paper showed that the rate of sIPSCs significantly increases throughout VIP-IN development [32], thus leading to a potential influence of sIPSCs on the estimation of *q*, especially in the P30 + age group. However, even at P30 +, the sIPSC rate is only 3 Hz and we therefore decided to neglect a putative contribution of sIPSCs to the estimation of *q.*

Secondly, our immunohistological investigations revealed an increase in putative inhibitory presynaptic structures as demonstrated by an increase in the perisomatic VGAT expression by P15, from which point on it remained on a plateau (Suppl. Figure 1). A developmental increase in VGAT was demonstrated by a study performing immunoblots on the developing rodent somatosensory cortex [49]. In agreement with our results, VGAT expression gradually increased until P15 and then remained constant until adulthood. This implies that neurons of the rodent somatosensory cortex follow a similar developmental trajectory in terms of the VGAT expression, which appears to be independent of the neuronal subtype.

Moreover, other GABAergic interneuron subtypes inhibit different subcellular compartments of their target cell. For instance, PV-expressing interneurons inhibit the perisomatic region, while SST-positive interneurons preferably target the dendrites [23, 50–53]. This was also demonstrated for the inhibition mediated by PV- and SST-positive interneurons towards VIP-INs of the adult mouse barrel field [23]. Since we investigated the VGAT expression in the perisomatic region of VIP-INs, this allows us to speculate that the observed increase in putative inhibitory presynaptic structures may mainly originate from PV-positive interneurons. More in particular, these inhibitory projections may mainly come from PV-INs residing in L4, since they have their highest abundance within this layer of the barrel field [24]. In addition, by providing feedforward inhibition [54], PV-positive cells of L4 undergo a phase of critical period plasticity until P14, influencing the tuning of L2/3 pyramidal neurons [55]. The investigated inhibitory projections to VIP-INs may also underlie such a phase of critical period plasticity, presumably important for the appropriate execution of active whisking.

### Precise inhibitory signal transmission via an accelerated vesicle replenishment

As the release probability increases, this should lead to a stronger presynaptic depression upon high network activity. However, we show that an acceleration of vesicle replenishment can elevate the steady-state phase, thus preventing an expected synaptic depression (Fig. 3). This is in line with other publications investigating vesicle replenishment and synaptic depression at hippocampal synapses [56, 57].

Interestingly, our data show that vesicle replenishment gradually accelerates between P9 and P30 +, while the release probability already increases rather stepwise by P15. This would imply that synaptic depression would be stronger at P15. However, the steady-state eIPSC remains at similar levels as compared to the P9 group (Fig. 3C). One reason for this might be that, despite the increase in the release probability being statistically significant, the total increase is less than 20%. The vesicle replenishment at P15 can therefore still be fast enough to prevent an increase in synaptic depression at this time point.

### Maturation towards a strong and precise signal transmission for a stimulus-locked inhibition

Analysis of asynchronously released vesicles demonstrates a significant decrease by reaching adulthood (Fig. 4B). Moreover, this decrease in ASR occurs in favour of an increase in stimulus-locked vesicle release (Fig. 4C). These results are in line with the maturation of inhibitory connections to neurons of the superior colliculus [40] and of the rat brain stem [58].

ASR is believed to depend on the [Ca^2+^] in the presynaptic bouton [35]. Presynaptic Ca^2+^-signalling at earlier time points may differ from that in adult stages. Indeed, Ca^2+^-homeostasis of the presynaptic inhibitory partner needs to undergo maturational processes to not only support a stimulus-locked vesicle release even at high network activity but also transmit this activity. Such maturational processes involve Ca^2+^-sequestration, Ca^2+^-release, and Ca^2+^-buffering. This may influence vesicle release and the replenishment rate, so that a stimulus-locked vesicle release can be executed in a high temporal resolution [59–61]. In the adult barrel field, L2/3 VIP-INs receive strong innervation from PV-INs, presumably mainly originating from L4 [23, 24]. PV itself is a Ca^2+^-buffering protein, and the corresponding interneurons in the somatosensory cortex start to show an immunoreactivity from around P14 on, which lasts until reaching adulthood [62]. This allows us to speculate that the shift from asynchronous to stimulus-locked vesicle release may depend on the expression of PV of the presynaptic partner. In line with this, past studies could demonstrate that the expression of Ca^2+^-buffering proteins such as parvalbumin or calbindin highly influences the short-term plasticity of inhibitory synapses [63–65].

It was shown that ASR is a mechanism to maintain inhibition despite a depletion of the RRP [35, 42, 66]. Additionally, an asynchronous vesicle release upon high network activity leads to a tonic inhibition, while a synchronous vesicle release leads to a phasic inhibition [35].

This also applies to our results in the following context: high network activity was simulated in our slices by applying a high-frequency stimulation, by which the RRP was emptied. We could show that in mice at the age of P9, when vesicles are asynchronously released, the inhibitory connections deplete faster, as indicated by a larger synaptic depression (Fig. 3C). This suggests that at this age, inhibition is mostly mediated by tonic, temporally imprecise vesicle release. Later in development, at the age of P30 +, a stimulus-locked vesicle release dominates, indicating that the activity pattern of synaptic inputs determines the temporal pattern of inhibition. This may be functionally relevant for a timely precise inhibition of VIP-INs so that active whisking can be performed in the appropriate frequency of ca. 10 Hz.

### Functional implications

Our present study shows that inhibitory connections from L4 to L2/3 VIP-INs in the barrel cortex undergo different maturational processes to provide a strong and temporally precise signal transmission. Consequently, VIP-INs can be precisely inhibited, leaving the opportunity of the presynaptic inhibitory partner to define temporal windows. Here, VIP-INs can integrate incoming excitation mediated by long-range projections from M1 and may disinhibit downstream pyramidal neurons, depending on their preferred downstream target. In addition, the definition of these temporal windows can be executed in an appropriate temporal resolution, necessary for performing active whisking at adequate frequencies.

### Limitations

This study investigated the maturation of inhibitory connections from L4 onto L2/3 VIP-INs by focusing on developmental changes in functional properties on the presynaptic side from an electrophysiological point of view. VIP-INs reflect a heterogeneous subpopulation of GABAergic INs, inter alia depicting different electrophysiological properties, morphologies, and preferred downstream targets [12, 67, 68]. This functionally involves the different VIP-IN subtypes in distinct inhibitory or disinhibitory circuitries [12], likely controlling network activity via different inhibitory operations. However, this study did not differentiate between the different VIP-IN subtypes, e.g., by biocytin-filling and post hoc visualisation. Thus, our study can only speculate about how the maturation of the inhibitory contacts onto the different VIP-IN subtypes may affect network activity. As this study focused on functional changes in the synapses within the presynaptic inhibitory partner of VIP-INs, we provide a detailed description of these developmental trajectories. This can be used by future studies to set this in context with the different VIP-IN subtypes and the inhibition of their preferred downstream targets.

## Supplementary Information

Below is the link to the electronic supplementary material.

ESM 1 (DOCX 566 KB)

## Data Availability

No datasets were generated or analysed during the current study.
