# Supplementary material for: Presynaptic maturation of inhibitory connections onto vasoactive intestinal polypeptide-expressing GABAergic interneurons in the mouse barrel field
Source: Pflugers Arch. 2025 Jun 25;477(8):1089–101. doi: 10.1007/s00424-025-03101-8 (PMC12310910; doi:10.1007/s00424-025-03101-8)
Supplement: Supplementary file 1 — (DOCX 566 KB) [file 424_2025_3101_MOESM1_ESM.docx]

**Supplementary Information (S1)**

**Fort the article:**

**Presynaptic maturation of inhibitory connections onto vasoactive intestinal polypeptide-expressing GABAergic interneurons in the mouse barrel field**

**Clara A. Simacek^1^, Sergei Kirischuk^1^, Thomas Mittmann^1^**

^1^Institute for Physiology, University Medical Centre of the Johannes Gutenberg University Mainz, Germany

**Submitted to the journal:** Pflügers Archiv – European Journal of Physiology

**Corresponding author:** Prof. Thomas Mittmann, [mittmann@uni-mainz.de](mailto:mittmann@uni-mainz.de)


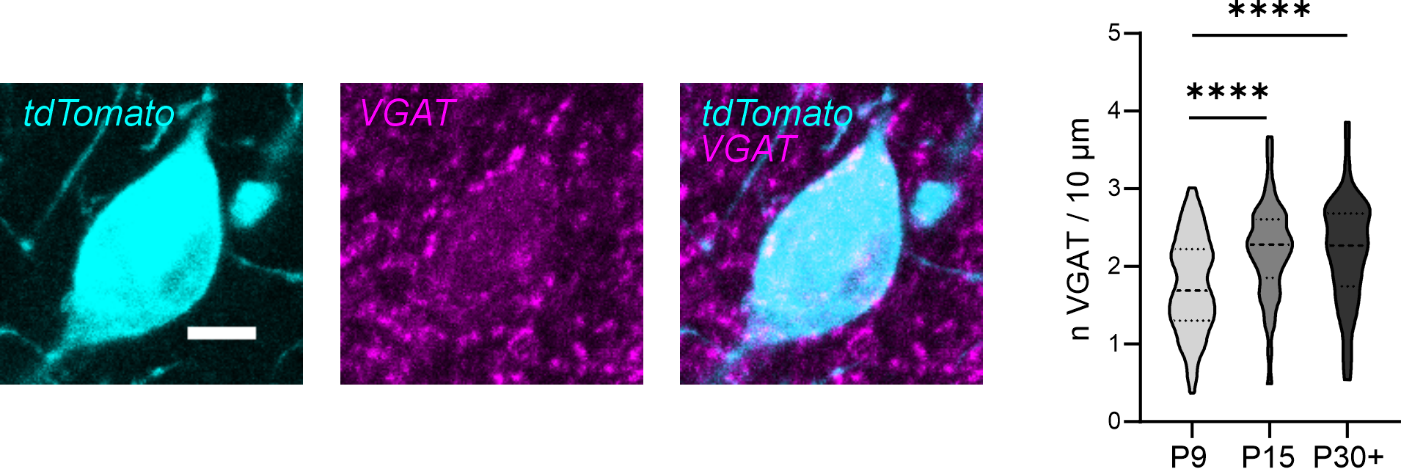


**Suppl. Figure 1 Increase in the number of VGAT-positive puncta on the somas of developing VIP-INs.** Left: Example immunohistological stainings of VIP-INs for tdTomato (left), VGAT (middle) and the two merged channels (right) at P30+. Right: Violin plots showing a significant increase in the number of VGAT-positive puncta between P9 and P15 from 1.8 ± 0.1 to 2.2 ± 0.1 (One-way ANOVA with Dunn’s post hoc test, F (2, 262) = 13.75, p < 0.0001), but not between P15 and P30+ (2.2 ± 0.1 for P30+, p = 0.8093) Number of cells: 80 – 99. **** p < 0.0001. Violin plots indicating minimum and maximum values, mean and SD.
